# The Economy of Child-Saving

**Published:** 1921-05-28

**Authors:** 


### The Economy of Child-Saving.

At the annual meeting of the National Baby Week Council, held at the Armitage Hall on Tuesday, May 24, Dr. Eric Pritchard, who was in the chair, read a message from the Queen, in which she expressed her pleasure in learning that the Council will celebrate its fifth anniversary in July, and sent her best wishes for its continued good works. Dr. Mabyn Read, Medical Officer for Worcester, gave some interesting details of the life of the people, and its effect on infant mortality. He finds that the chief industry, glove-making, which is largely carried on at home, does not affect the child death-rate. When he first went to Worcester in 1890 the infant mortality rate was 208 per thousand; in 1920 it was 67 per thousand. This he attributes largely to the work of the health visitors in visiting the people in their homes. Miss Eleanor Rathbone, confessing that she could not speak from a practical point of view regarding infants, as she was not a mother, a doctor, or a nurse, emphasised the fact that it is in the early days of married life when the children are young that privation and poverty are most disastrous in their effects. It is poor economy to pay a "family" living wage to workers, 52 per cent. of whom are bachelors with no family. The bachelor wastes his money; the father of more than three children cannot give them sufficient bread. She suggested that the system of paying a wage according to position is wrong, and that the wage should be adjusted according to the needs of the family. Dr. Leonard Hill urged the economy of giving the child plenty of fresh air and sunshine to keep him healthy, and not only providing it when he is sick. He maintained that "dark heat" such as is produced by radiators or hot-air pipes is injurious, because it only reaches the skin, and dries it, and never penetrates to the blood. The luminous rays of an open fire, like those of the sun, are absorbed by the blood, which in turn heats the whole body. Professor Collis, of Cardiff, also urged the true economy of money spent to ensure health. The multiplication of health visitors would mean a great saving of money and life to the nation.

